# Homozygosity mapping provides supporting evidence of pathogenicity in recessive Mendelian disease

**DOI:** 10.1038/s41436-018-0281-4

**Published:** 2018-10-03

**Authors:** Matthew Neil Wakeling, Thomas William Laver, Caroline Fiona Wright, Elisa De Franco, Karen Lucy Stals, Ann-Marie Patch, Andrew Tym Hattersley, Sarah Elizabeth Flanagan, Sian Ellard, DDD Study

**Affiliations:** 1 Institute of Biomedical & Clinical Science, University of Exeter, Exeter, UK; 2 Department of Molecular Genetics, Royal Devon & Exeter NHS Foundation Trust, Exeter, UK; 3 QIMR Berghofer, Herston, Queensland Australia

**Keywords:** variant interpretation, ACMG guidelines, Mendelian disease, recessive disease, genetic diagnosis

## Abstract

**Purpose:**

One of the greatest challenges currently facing those studying Mendelian disease is identifying the pathogenic variant from the long list produced by a next-generation sequencing test. We investigate the predictive ability of homozygosity mapping for identifying the regions likely to contain the causative variant.

**Methods:**

We use 179 homozygous pathogenic variants from three independent cohorts to investigate the predictive power of homozygosity mapping.

**Results:**

We demonstrate that homozygous pathogenic variants in our cohorts are disproportionately likely to be found within one of the largest regions of homozygosity: 80% of pathogenic variants are found in a homozygous region that is in the ten largest regions in a sample. The maximal predictive power is achieved in patients with <8% homozygosity and variants >3 Mb from a telomere; this gives an area under the curve (AUC) of 0.735 and results in 92% of the causative variants being in one of the ten largest homozygous regions.

**Conclusion:**

This predictive power can be used to prioritize the list of candidate variants in gene discovery studies. When classifying a homozygous variant the size and rank of the region of homozygosity in which the candidate variant is located can also be considered as supporting evidence for pathogenicity.

## Introduction

The advent of high-throughput next-generation sequencing has been a boon to the study of Mendelian disease. It is now possible to screen thousands of genes in a single test. However, this generates an extensive list of variants. One of the greatest challenges currently facing those studying Mendelian disease is identifying the pathogenic variant amongst the myriad of other variants.^1^ To help with this task the American College of Medical Genetics and Genomics (ACMG) has developed guidelines^2^ for variant interpretation, providing a process for classifying variants using all different types of potential available evidence.

Searching for shared regions of homozygosity between affected individuals has been used to identify genes causing recessive Mendelian diseases.^3^ Identifying target genes within shared regions of homozygosity is a critical step in consanguineous families with recessive disorders.^4^ Regions of homozygosity are created when identical-by-descent haplotypes are inherited from parents. A homozygosity map can be generated directly from next-generation sequencing data, identifying regions likely to contain the causative variant.^5^

The number and size of homozygous regions within an individual’s genome is influenced by ancestral population effects and recent consanguineous events. It is important to differentiate the two cases as disease-causing variants are likely to be in regions of recent homozygosity; variants in ancestral regions of homozygosity have been exposed to selection in a homozygous state for sufficient time for selection to act on them. Ancestral regions of homozygosity are likely to be smaller, less than a megabase, whereas homozygous regions that are the result of recent consanguinity tend to be multiple megabases in length.^6^ Thus we would expect variants that cause recessive Mendelian disease to be contained in the largest regions of homozygosity.

To test the hypothesis that homozygous pathogenic variants are more likely to be found in the largest regions of homozygosity in a sample, we used a data set of 99 consanguineous patients with previously identified homozygous pathogenic variants. We then replicated our findings in two further cohorts, with 17 and 63 patients respectively.

## Materials and methods

### Cohort descriptions

Our discovery cohort consisted of patients referred to the molecular genetics department at the Royal Devon and Exeter Hospital for genetic testing for neonatal diabetes (NDM) or hyperinsulinemic hypoglycemia (HH). Samples were sequenced on a targeted gene panel test for monogenic diabetes and HH.^9^ 99 consanguineous patients were diagnosed as having a homozygous pathogenic variant.

We replicated our findings in two further cohorts: first, consanguineous patients with severe pediatric disorders where exome sequencing identified 17 homozygous pathogenic variants; and second, 63 consanguineous children from the Deciphering Developmental Disorders (DDD) study^7,8^ with a pathogenic or likely pathogenic homozygous variant identified using trio exome sequencing and shared via DECIPHER.^10^

Patients were defined as consanguineous if more than 1.5% of their genome was covered by homozygous regions >3 Mb. This is the expected percentage of homozygosity for offspring of second cousin marriages.^11^ Levels of homozygosity were similar between cohorts: discovery cohort mean 8.7% (SD 4.5%), severe pediatric disorders cohort 8.8% (6.6%), DDD cohort 9.2% (4.5%).

Informed consent was obtained at referral. See Supplementary Information for details on consent and statistics.

### Homozygosity mapping

For our discovery cohort, regions of homozygosity were detected directly from the targeted sequencing data using SavvyHomozygosity, which uses off-target reads.^12,13^ For the two replication cohorts, regions of homozygosity were calculated from VCF files using SavvyVcfHomozygosity.^12,13^ The pathogenic variants in our samples were discovered independently of the regions of homozygosity mapping; they were not used to guide variant discovery.

## Results

### 79% of pathogenic variants are found in a homozygous region that is in the ten largest regions

In our discovery cohort we found that the largest regions of homozygosity in each sample were more likely to contain the pathogenic variant. In fact, the rank (receiver operator characteristic [ROC] area under the curve [AUC] 0.666), size (AUC 0.627), and relative size (size of homozygous region divided by size of the largest region in the sample) (AUC 0.668) all have predictive power (Supplementary Figure 1A). 79% of pathogenic variants are found in the ten largest homozygous regions in a sample. 87% of pathogenic variants are found in a homozygous region >5 Mb. 84% of pathogenic variants are found in a homozygous region no more than five times smaller than the largest region. The mean size of the homozygous regions in our samples is 18.9 Mb (SD 15.1 Mb) while 89.7% of homozygous regions are >5 Mb. The predictive ability of the combined metrics is greater than any individual measure (AUC 0.684).

### The largest regions have predictive value over and above the proportion of homozygosity they account for

**Figure 1:**
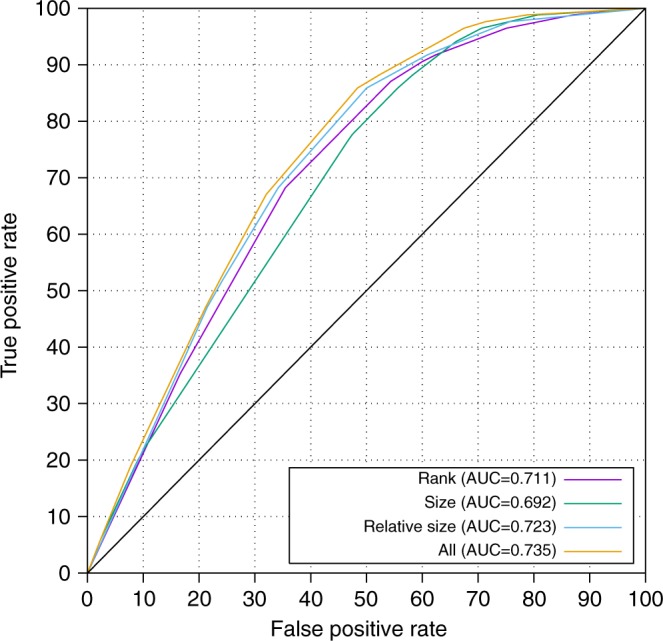
**Rank, size, and relative size have predictive power.** The receiver operator characteristic (ROC) curve for our combined data set (discovery cohort plus replication cohorts, excluding samples with homozygosity >8% and variants within 3 Mb of a telomere) demonstrates that there is positive predictive value for each of rank, size, and relative size, with the highest predictive value coming when these are combined

Figure 1 and Supplementary Figure 2 demonstrate that the causative variant is disproportionately likely to be in a large region, over and above the proportion of homozygous bases the region accounts for. For example, in our discovery cohort 79% of pathogenic variants are in the ten largest regions but these only account for 55% of homozygous bases. The number of pathogenic variants in the 50% of bases accounted for by the largest regions of homozygosity is significantly higher than the number of pathogenic variants in the 50% of bases from the smallest regions (*P* = 5.5 × 10^-5^). We have sufficient power to detect this effect: a minimum of 51 samples is required to detect the proportion with 80% power and *P* = 0.05. This pattern is demonstrated by the ROC curve in Supplementary Figure 1A.

### Homozygous region rank and size have predictive power in replication cohorts

We replicated our findings in two independent cohorts. The rank, size, and relative size of the homozygous regions all have predictive power in both replication cohorts (Supplementary Figures 1B and 1C). When we combine all three data sets the AUC is 0.630 for rank, 0.613 for size, 0.643 for relative size, and 0.654 combining all three metrics (Supplementary Figure 1D). In the combined data set 80% of pathogenic variants are found in the ten largest regions.

### Excluding samples with homozygosity >8% and variants within 3 Mb of a telomere improves predictive power

**Figure 2:**
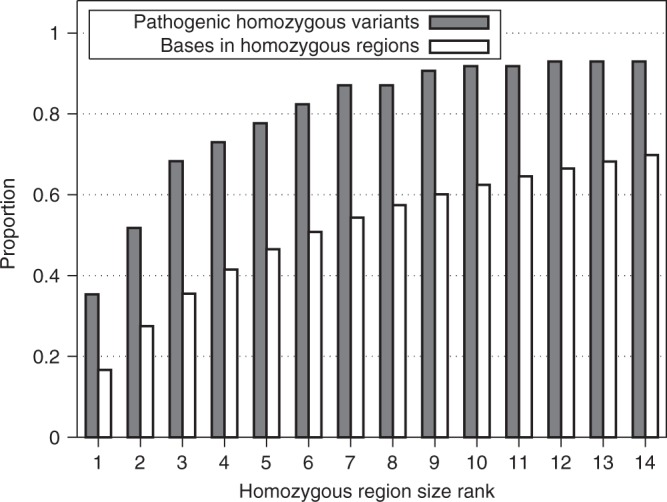
**The largest regions of homozygosity contain more pathogenic variants than would be expected from the proportion of homozygous bases the regions account for.** Results shown for our combined data set (discovery cohort plus replication cohorts), excluding samples with homozygosity >8% and variants within 3 Mb of a telomere. The solid bars represent the cumulative proportion of homozygous pathogenic variants that are within regions of that rank or larger while the hollow bars represent the cumulative number of bases within homozygous regions of that rank or larger. AUC is the area under the curve

We investigated the characteristics of those samples where the causative variant was not in one of the ten largest regions: these had a higher amount of homozygosity (mean 11.9 vs. 8.3%). Additionally genes near telomeres were more likely to have causative variants that were not in the ten largest regions (eight variants within 3 Mb of a telomere, only one in the ten largest regions, *P* = 0.000055, Fisher's exact test). If we only include samples with <8% homozygosity and exclude variants within 3 Mb of a telomere the AUC increases to 0.735 and 92% of causative variants are in one of the ten largest homozygous regions (Figure 2).

### Using rank and relative size of the homozygous region to guide variant interpretation

Using rank alone to evaluate pathogenicity has predictive power, but using multiple metrics improves on this. Supplementary Table 1 provides a homozygosity rank (HR) score for homozygous regions based on the rank and relative size of our combined data set (excluding samples with homozygosity >8% and variants within 3 Mb of a telomere). The HR score is the percentage of bases in homozygous regions that are smaller than the one under consideration. Ninety-two percent of causative variants are in a homozygous region with a HR score of 42 or more; this threshold can be used in the routine assessment of novel variants.

## Discussion

### Presence of a variant in a large region of homozygosity has predictive power

We demonstrate in our discovery cohort that the rank, size, and relative size of homozygous regions have predictive power for whether a variant is causative. We replicated this pattern in two independent cohorts.

We would expect the causative variant to be in the largest regions of homozygosity because these have been formed by recent consanguineous events.^6^ Smaller regions are present in the population from ancestral events and have thus been in the population for longer; this means they have been exposed to selection pressures for longer, thus are less likely to contain disease-causing variants. We expect to see enrichment of pathogenic variants in the largest homozygous regions in all recessive Mendelian disorders where the disease is severe enough to strongly affect reproductive fitness.

### Presence of a variant in one of the ten largest regions of homozygosity is supporting evidence for pathogenicity

The ACMG guidelines^2^ incorporate different types of evidence into the overall classification: population frequency data, in silico predictions, functional data, and cosegregation of the variant with the disease within the family. We have demonstrated that a variant being within a large homozygous region has predictive power as to the pathogenicity of the variant. The data used by this test is uncorrelated with other predictors of pathogenicity so can be used in combination. We therefore suggest that the presence of a homozygous variant in one of the ten largest regions of homozygosity could to be used as supporting evidence in the context of variant classification using the ACMG guidelines.

### Limitations

The samples for this study are from multiple global populations, which could be a confounding factor as different populations are known to have different patterns of homozygosity.^6^ We also observed that in samples with greater levels of homozygosity predictive power was reduced. However, there is predictive power even in samples with very high (>8%) levels of homozygosity and we suggest that the biological principle should be generally applicable across individuals and populations—that the causative homozygous variant will tend to be in a larger homozygous region, because these are the result of recent consanguineous events. This metric should be applicable for all consanguineous patients—consanguinity (homozygosity >1.5%) can be determined from sequencing data and does not need to be known *a priori*.

The predictive power of homozygous regions should be agnostic to the method used to call the regions; however, certain areas of the genome are harder to sequence and thus contain more false heterozygous variants, which have the potential to artificially break up large regions of homozygosity. This can be reduced by using only variants that are in Hardy–Weinberg equilibrium and allowing some heterozygous variants within homozygous regions.

We observed that causative variants close to telomeres were less likely to be within the ten largest regions of homozygosity. We hypothesize that proximity to the end of the chromosome restricts the size of the homozygous region; this is an application of the inspection paradox^14^ (Supplementary Information). Thus we caution against using this metric to exclude variants within 3 Mb of a telomere.

### This test only provides supporting evidence for pathogenicity

Within our data set, some of the pathogenic variants were not present in a large homozygous region; this is likely caused by small community effects and founder mutations, as well as the effect of proximity to a telomere. It is therefore important to remember that the presence of a variant outside of a large homozygous region does not prove it is benign just as the presence of a variant in one of the largest regions of homozygosity does not provide conclusive evidence of pathogenicity. It does however provide additional complementary evidence with a similar predictive power (overall AUC 0.654 rising to 0.735 excluding samples with homozygosity >8% and variants within 3 Mb of a telomere) to widely used tools such as SIFT (AUC 0.631–0.848) and PolyPhen (AUC 0.596–0.859)^15^.

### Homozygosity mapping guides gene discovery

We can apply our results to prioritize the list of candidate variants in gene discovery studies. For example, 80% of pathogenic variants are found in a homozygous region that is in the ten largest regions but only 61% of homozygous bases fulfill the same criteria. Using such a prioritization enriches the remaining regions for pathogenic variants. This is of particular value for gene discovery within consanguineous cohorts without multiple affected members in a single family to narrow down target regions.

### Conclusion

In conclusion, the size, rank, and relative size of the homozygous region a variant is found in provides evidence of its likely pathogenicity. 92 percent of pathogenic variants are found in the ten largest regions of homozygosity (excluding samples >8% homozygosity and variants within 3 Mb of a telomere). We suggest this criterion could be used in the context of the ACMG guidelines as a potential source of supporting evidence for variant pathogenicity.

## Electronic supplementary material

Supplementary Figure 1

Supplementary Figure 2

Supplementary Information

Supplementary Table
