# Correction: Homozygosity mapping provides supporting evidence of pathogenicity in recessive Mendelian disease

**DOI:** 10.1038/s41436-018-0357-1

**Published:** 2018-11-16

**Authors:** Matthew Neil Wakeling, Thomas William Laver, Caroline Fiona Wright, Elisa De Franco, Karen Lucy Stals, Ann-Marie Patch, Andrew Tym Hattersley, Sarah Elizabeth Flanagan, Sian Ellard, DDD Study

**Affiliations:** 1 Institute of Biomedical & Clinical Science, University of Exeter, Exeter, UK; 2 Department of Molecular Genetics, Royal Devon & Exeter NHS Foundation Trust, Exeter, UK; 3 QIMR Berghofer, Herston, Queensland Australia

Correction to: *Genetics in Medicine*: doi: 10.1038/s41436-018-0281-4

The original version of this Article contained an error in the top left of Figure 2: the number 1 on the *y*-axis had been changed to 0 during the typesetting process. This has now been corrected in both the PDF and HTML versions of the Article.

In addition, this Article was originally published without the accompanying Supplementary Table 1. This file is now available in the HTML version of the Article; the PDF was correct from the time of publication.

