# Supplementary figures and images for: Homozygosity mapping provides supporting evidence of pathogenicity in recessive Mendelian disease
Source: Genet Med. 2018 Oct 3;21(4):982–6. doi: 10.1038/s41436-018-0281-4 (PMC6330071; doi:10.1038/s41436-018-0281-4)

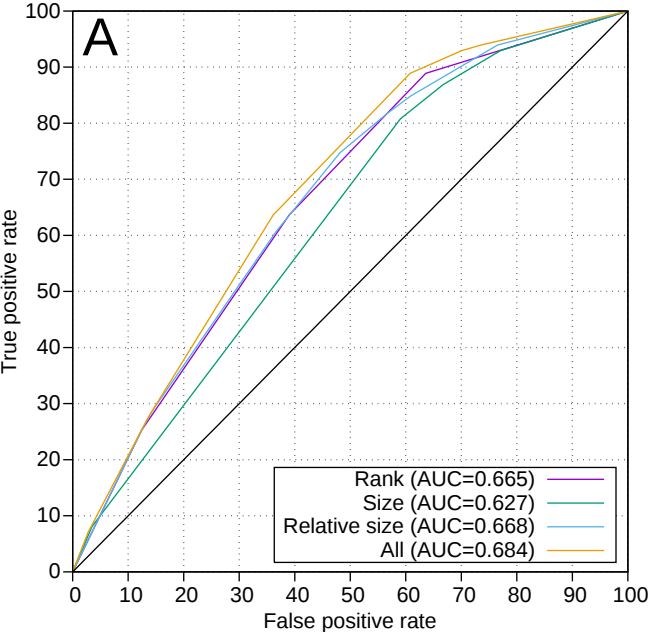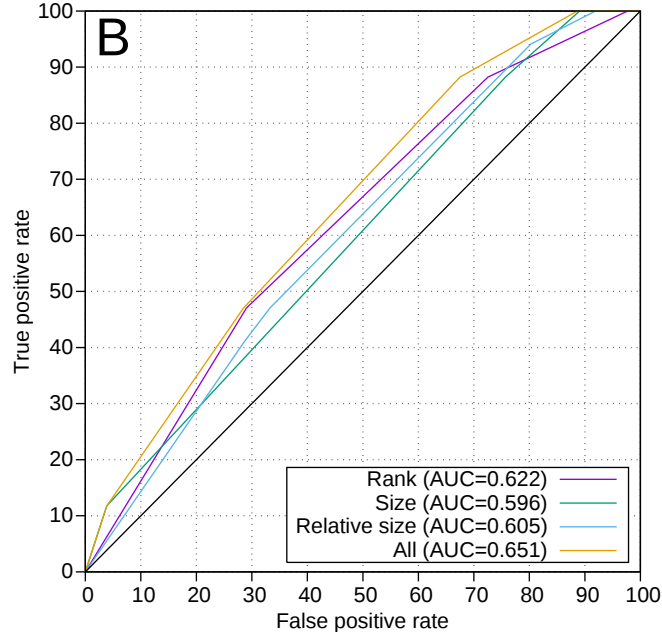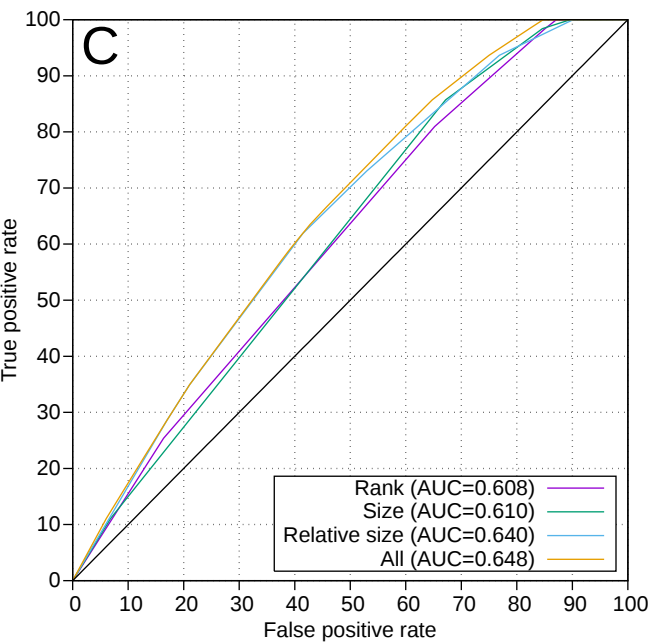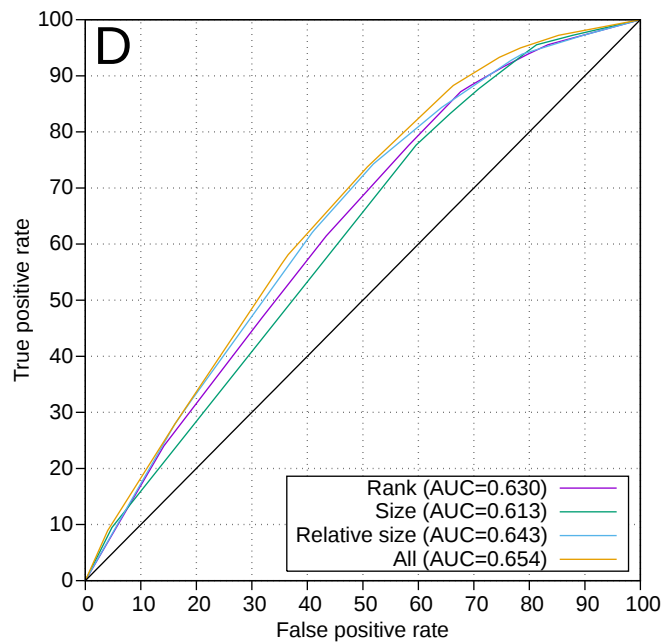

Supplement: Supplementary file 1 — Supplementary Figure 1 [file 41436_2018_281_MOESM1_ESM.pdf]

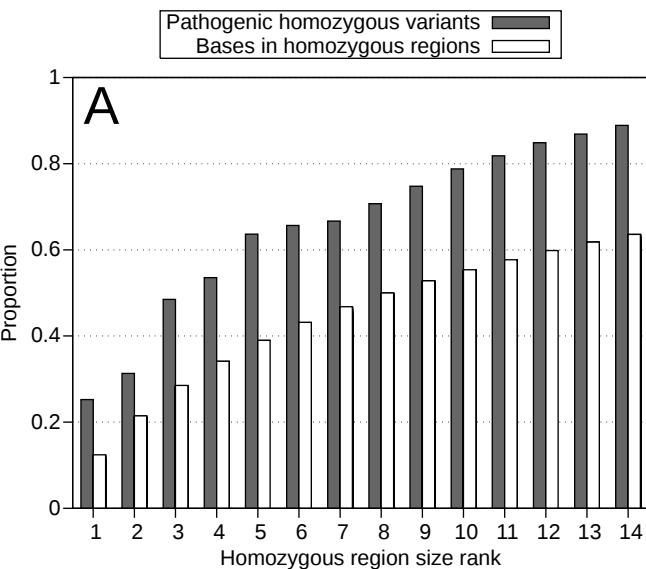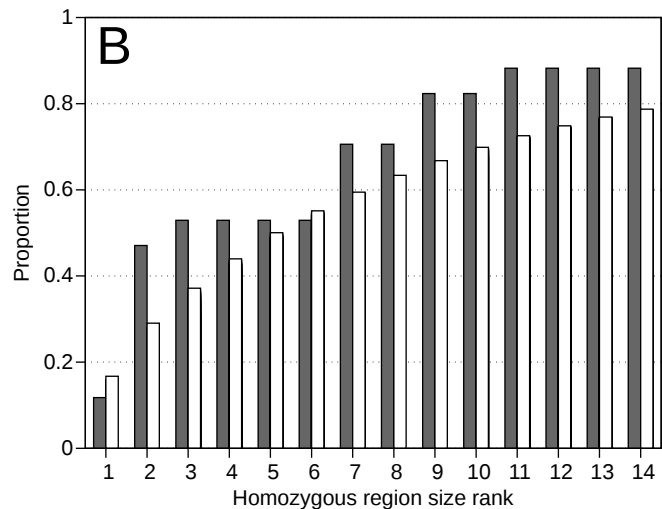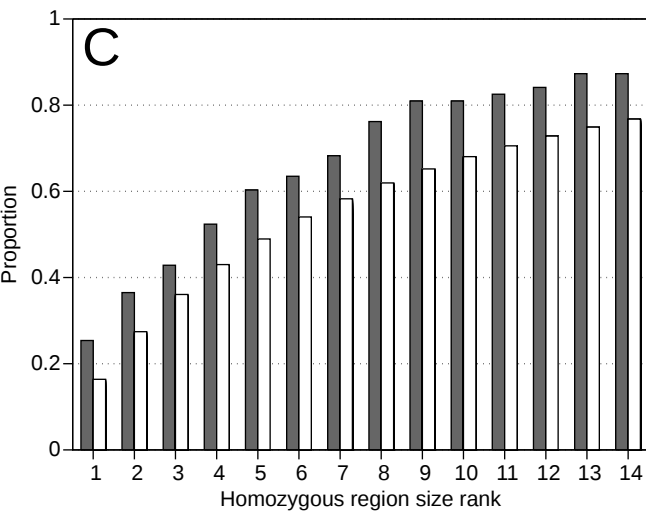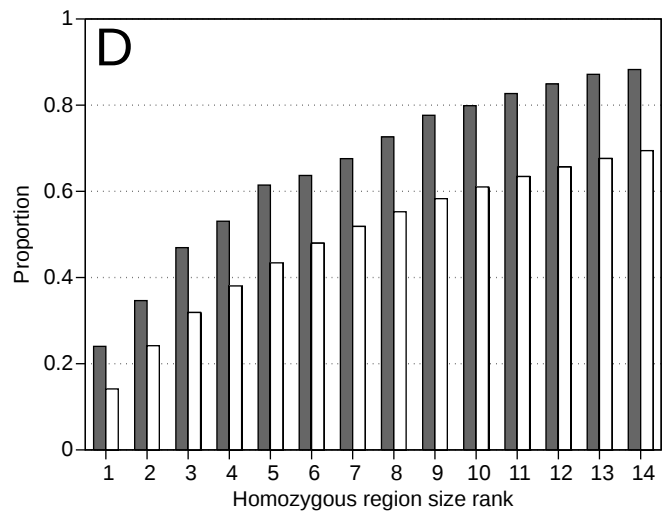

Supplement: Supplementary file 2 — Supplementary Figure 2 [file 41436_2018_281_MOESM2_ESM.pdf]
