# Supplementary material for: Homozygosity mapping provides supporting evidence of pathogenicity in recessive Mendelian disease
Source: Genet Med. 2018 Oct 3;21(4):982–6. doi: 10.1038/s41436-018-0281-4 (PMC6330071; doi:10.1038/s41436-018-0281-4)
Supplement: Supplementary file 3 — Supplementary Information [file 41436_2018_281_MOESM3_ESM.docx]

# Homozygosity mapping provides supporting evidence of pathogenicity in recessive Mendelian disease

# Supplementary information

**Materials and Methods**

**Consent and ethics**

Informed consent was obtained for all samples at the time of referral for genetic testing. The DDD study was approved by UK Research Ethics Committee 10/H0305/83 (granted by the Cambridge South REC, and GEN/284/12 granted by the Republic of Ireland REC). The research team acknowledges the support of the National Institute for Health Research, through the Comprehensive Clinical Research Network. This study uses DECIPHER (<https://decipher.sanger.ac.uk>), which is funded by the Wellcome. Anonymised data was used for the analysis.

**Data access statement**

Due to the confidential nature of some of the research materials supporting this publication not all of the data can be made accessible to other researchers. Please contact the corresponding author for more information.

**Statistics**

True positive rate (TPR): The proportion of pathogenic variants that are correctly detected.

False positive rate (FPR): The proportion of benign variants that are incorrectly detected as positive. This is the proportion of bases in homozygous regions that fulfil the criteria, given we can assume that the number of variants is proportional to the size of the homozygous region, and that most of the variants are not pathogenic.

A ROC curve is constructed by varying the rank/size/relative size threshold (for example only rank 1, ranks 1 and 2 etc), finding the TPR and FPR for each configuration, and plotting as a point on the graph. The line is then drawn around the points. The area under the curve is calculated by taking each line section, calculating the area of the right trapezoid under each one, and summing the areas.

We used a binomial distribution to calculate the significance of the higher count of pathogenic variants in the 50% of bases covered by the largest regions of homozygosity compared to the count of pathogenic variants in the 50% bases covered by smaller regions of homozygosity.

**Discussion**

**The inspection paradox**

Regions of homozygosity near a telomere are significantly smaller than regions of homozygosity away from a telomere. This does not require any increase in the rate of crossover during meiosis, but we suggest this is due to the inspection paradox[14], which is a statistical effect. If a random position is chosen near the middle of a chromosome, and this is within a region of homozygosity, then this region of homozygosity can extend both towards the p arm and the q arm of the chromosome until the crossover point is reached. In contrast, if a position is chosen at the telomere, then the region of homozygosity can only extend towards the centromere, and is statistically half the size.

**Supplementary figure legends**

Supplementary Figure 1: Rank, size and relative size have predictive power. Part A shows the data from our discovery cohort while parts B (severe paediatric disorders cohort) and C (DDD cohort) show the data from our replication cohorts. Part D shows the combined set of samples. The ROC curves demonstrate that for each of the cohorts examined there is positive predictive value for each of rank, size and relative size, with the highest predictive value coming when these metrics are combined.

Supplementary Figure 2: The largest regions have predictive value over and above the proportion of homozygosity they account for. Part A shows the data from our discovery cohort while parts B (severe paediatric disorders cohort) and C (DDD cohort) show the data from our replication cohorts. Part D shows the combined set of samples. The solid bars represent the proportion of homozygous pathogenic variants which are accounted for by regions of that rank or larger while the hollow bars represent the number of bases accounted for by homozygous regions of that rank or larger.

Supplementary Table 1: The Homozygosity Rank Score (HR score) of homozygous regions based on the ranks and relative sizes from our data. This provides a look-up table for researchers and clinical scientists to look up the rank and relative size of the homozygous region their putative pathogenic variant to guide variant interpretation. The rank and relative size of the homozygous region in which a putative pathogenic variant is in can be looked up in this table; the score will then inform the likelihood of a homozygous region of that score or better containing the pathogenic variant.
